# Silent Ovary with a Loud Hormonal Signal: A Case Report and Literature Review of an Androgen-Secreting Stromal Luteoma

**DOI:** 10.3390/jcm15197506

**Published:** 2026-09-27

**Authors:** Milina Tančić-Gajić, Radomir Aničić, Gorana Nikolić, Marija Miletić, Ljiljana Popović, Milica Jovanović, Taisa Bojović, Anđela Bošković, Teodora Čavić, Svetlana Vujović

**Affiliations:** 1 Faculty of Medicine, University of Belgrade, Dr Subotića 8, 11000 Belgrade, Serbia; 2 Clinic for Endocrinology, Diabetes and Metabolic Diseases, University Clinical Centre of Serbia, Dr Subotića 13, 11000 Belgrade, Serbia; 3 Clinic for Gynecology and Obstetrics “Narodni Front”, Kraljice Natalije 62, 11000 Belgrade, Serbia; 4 Institute of Pathology “Dr. Ðorđe Joannović”, Faculty of Medicine, University of Belgrade, 11000 Belgrade, Serbia

**Keywords:** ovarian neoplasms, steroid cell tumor, stromal luteoma, Postmenopause, hyperandrogenism, virilism, laparoscopy

## Abstract

**Background**: Less than 0.1% of ovarian cancers are ovarian steroid cell tumors, which are very uncommon sex cord-stromal neoplasms. A rare and usually benign form, stromal luteomas primarily affect postmenopausal women. These tumors can have major hormonal consequences despite their small size. The most common symptom is estrogen excess; androgen release with virilization is uncommon and might be challenging to spot, especially when imaging is subtle. **Case Presentation**: A 57-year-old postmenopausal woman presented with a three-year history of escalating hyperandrogenism, which included clitoromegaly, frontotemporal hair thinning, deeper voice, and quickly deteriorating hirsutism. Laboratory analyses revealed significantly elevated serum testosterone levels, reaching the male reference range, accompanied by reduced gonadotropins. Androstenedione and DHEAS levels were within normal ranges. Cushing’s syndrome and congenital adrenal hyperplasia were excluded. Although transvaginal color Doppler ultrasonography revealed asymmetric ovarian enlargement of the left ovary, initial imaging revealed neither ovarian nor adrenal tumors. The patient underwent a bilateral oophorectomy performed laparoscopically. A 1.5-cm steroid cell tumor of the left ovary, compatible with stromal luteoma, was verified by histopathology and immunohistochemistry. Following surgery, testosterone levels rapidly returned to normal, while virilizing features gradually regressed. **Conclusions**: This example highlights how, even in cases when initial imaging is unremarkable, postmenopausal women with rapid virilization and noticeably higher testosterone levels should be evaluated for rare androgen-secreting ovarian tumors. A timely diagnosis requires a complete assessment of ovarian morphology and a thorough endocrine evaluation. Both biochemical abnormalities and clinical signs can be resolved with surgical excision, which offers a definitive treatment.

## 1. Introduction

The 2020 World Health Organization (WHO) Classification of Female Genital Tumors (5th edition) states that ovarian steroid cell tumors (SCTs) account for less than 0.1% of all ovarian tumors and are categorized under the sex cord-stromal tumor group [1,2]. Within this group, stromal luteomas represent an even rarer subset, comprising typically benign tumors that arise from ovarian stromal cells and are predominantly associated with postmenopausal women. Their capacity to release hormones can result in pronounced endocrine manifestations, although they are frequently clinically silent and only identified by accident. This unique clinical paradox is evident in the difference between low tumor burden and significant hormone action [3,4]. The most typical clinical manifestation of an androgen-secreting stromal luteoma is virilization, which is marked by symptoms that progress quickly, including clitoromegaly, temporal baldness, hirsutism, and voice deepening [5,6]. This stark contrast between the tumor’s small size and its potent endocrine activity creates a unique clinical scenario: a physically silent ovarian mass characterized by a marked biochemical and clinical signal [3]. This case study documents a case of a postmenopausal woman presenting with virilization due to a rare androgen-secreting ovarian stromal luteoma. It describes the diagnostic process in detail, starting with clinical suspicion and continuing with biochemical profiling, imaging, surgical excision, and conclusive histological confirmation.

## 2. Case Presentation

A 57-year-old Caucasian postmenopausal female reported significant frontal and temporal hair loss along with diffuse hair thinning that had been present for three years. Additionally, she observed a rapid increase in hair growth in androgen-sensitive areas, specifically the thighs, supra- and infra-umbilical abdomen, chest, chin, and upper lip. The patient reported menarche at age 14, followed by regular monthly cycles until menopause onset at age 52. Her obstetric history included two full-term deliveries. She had not been treated for reproductive issues or experienced infertility. However, she was diagnosed with polycystic ovarian syndrome (PCOS) throughout her reproductive years. Her long-term pharmacotherapy included ACE inhibitors, beta-blockers, metformin, and statins for hypertension, type 2 diabetes mellitus, and hyperlipidemia. At age 53, she underwent a hysterectomy due to uterine prolapse. She denied using anabolic/androgenic steroids, smoking, or drinking alcohol. Her family history was non-contributory. Central obesity (BMI 31 kg/m^2^, waist circumference 101 cm, hip circumference 103 cm) and considerable hirsutism (modified Ferriman–Gallwey score 17) were noted during the physical examination. Virilization symptoms included clitoromegaly, voice deepening, and androgenic alopecia (Ludwig stages II-2/III) (Figure 1).

There were no clinical signs of acromegaly or stigmata of Cushing’s disease. Hormonal analysis revealed significantly elevated serum testosterone levels, similar to those found in healthy adult males, along with a significantly higher free androgen index and gonadotropins that were inappropriately repressed for a postmenopausal woman. Serum estradiol level was marginally above the reference interval for postmenopausal women. Concentrations of serum 17-hydroxyprogesterone were within the normal range, excluding congenital adrenal hyperplasia caused by 21-hydroxylase deficiency. Notably, other circulating androgens, such as androstenedione and dehydroepiandrosterone sulfate (DHEAS), an androgen mostly produced by the adrenal glands, remained within the reference range (Table 1).

The 48-h 2 mg/day low-dose dexamethasone suppression test (LDDST) demonstrated appropriate suppression of cortisol but not testosterone, excluding autonomous cortisol excess and further suggesting an androgen-secreting tumor (Table 1). The patient was euthyroid (TSH 0.58 mIU/L, fT4 10.0 pmol/L). All measured tumor markers, including carcinoembryonic antigen (CEA), cancer antigen-125 (CA-125), human epididymis protein 4 (HE4), carbohydrate antigen 19-9 (CA 19-9), calcitonin, alpha-fetoprotein (AFP), neuron-specific enolase (NSE), and beta-human chorionic gonadotrophin (β-hCG), were within their respective reference ranges. The patient had a three-year history of gradually progressive hyperandrogenic symptoms, including diffuse hair thinning, androgen-dependent hair growth, and subsequent virilization. However, she did not seek medical evaluation during the initial period, allowing symptoms to progress gradually over time. Six months before referral to our center, the progression of hyperandrogenic features prompted an initial gynecological evaluation. Transvaginal ultrasonography performed at that time showed normal ovarian morphology. Due to the marked hyperandrogenism and a suspicion of an androgen-producing tumor, magnetic resonance imaging (MRI) of the abdomen and pelvis was subsequently performed during the same evaluation. MRI revealed no adrenal or ovarian masses; both ovaries were preserved, and the pelvic findings were consistent with postoperative changes following previous hysterectomy. The right ovary measured 24 × 12.2 mm and the left ovary measured 28 × 13 mm, with normal MRI appearance and no focal lesions.

Six months later, during evaluation at our center, repeat abdominal ultrasonography demonstrated no abnormalities of the adrenal glands or other abdominal structures. Transvaginal color Doppler imaging showed preserved and symmetrical bilateral ovarian artery flow; however, it revealed enlargement of the left ovary (36 × 16 × 32 mm; volume 11.5 cm^3^) compared with the right ovary (34 × 19 × 12 mm; volume 3.5 cm^3^).

Next, a laparoscopic bilateral oophorectomy was performed on the patient. The lesion was a well-circumscribed, 1.5-cm nodular proliferation located within the left ovarian parenchyma. It was not associated with the ovarian hilum. The right ovary showed no significant macroscopic changes. Under a microscope, the tumor appeared to be benign, consisting of sheets of luteinized cells with abundant eosinophilic cytoplasm and sporadic lipofuscin pigmentation. No mitotic activity or cellular atypia was present (Figure 2). Careful examination of the available sections did not demonstrate diffuse or multifocal proliferation of luteinized stromal cells in the surrounding ovarian parenchyma, nor was any stromal hyperthecosis identified. We did not identify Reinke crystals upon examination of the hematoxylin and eosin (H&E)-stained sections.

Immunohistochemical analysis revealed strong positivity for inhibin, calretinin, Melan-A, and CD99 (Figure 3), whereas chromogranin, synaptophysin, S-100 protein, and EMA were negative. The proliferative index, assessed by Ki-67, was below 1% (Figure 4). Oil Red O staining was not performed because the available material consisted of routinely formalin-fixed, paraffin-embedded tissue, and no fresh or frozen tissue was available for lipid staining.

These findings, together with the morphology and immunohistochemical profile described above, support the historical diagnosis of stromal luteoma. In accordance with current WHO terminology, the lesion falls within the category of ovarian steroid cell tumor, NOS (7). The term “stromal luteoma” is retained here as a historical and morphological designation. In the present case, the neoplastic cells demonstrated diffuse cytoplasmic positivity for calretinin, inhibin-α, Melan-A (MART-1), and CD99. Calretinin, a calcium-binding protein expressed in normal ovarian stromal and sex cord-derived cells, is a sensitive marker of sex cord-stromal differentiation and is typically expressed in stromal luteoma, supporting its ovarian stromal origin. Inhibin-α is a glycoprotein hormone produced by granulosa and luteinized stromal cells and represents one of the most informative immunohistochemical markers for ovarian sex cord-stromal tumors. Strong cytoplasmic positivity for inhibin-α supports the diagnosis of stromal luteoma and helps distinguish it from epithelial and metastatic ovarian neoplasms. Melan-A (MART-1), although originally recognized as a melanocytic marker, is also expressed in steroid-producing cells of the adrenal cortex and gonads. Melan-A positivity supports the diagnosis of stromal luteoma by indicating steroidogenic differentiation. CD99 is a transmembrane glycoprotein frequently expressed in sex cord-stromal tumors. Although not entirely specific, its membranous positivity interpreted in conjunction with calretinin, inhibin-α, and Melan-A expression further supports the diagnosis of stromal luteoma.

Conversely, the tumor was negative for chromogranin A, synaptophysin, S100 protein, and epithelial membrane antigen (EMA). Chromogranin A and synaptophysin are neuroendocrine markers, and their absence is inconsistent with neuroendocrine differentiation in tumors with overlapping morphological features. S100 protein is associated with neural crest-derived tissues and melanocytic lesions; therefore, negative staining helps exclude melanocytic and peripheral nerve sheath tumors from the differential diagnosis. EMA is an epithelial marker typically expressed in epithelial ovarian neoplasms, and its absence supports a non-epithelial origin consistent with stromal luteoma [7,8,9,10,11,12,13].

Overall, the combined immunophenotype—positive for calretinin, inhibin-α, Melan-A, and CD99, and negative for chromogranin A, synaptophysin, S100 protein, and EMA—together with the characteristic morphological features, strongly supports ovarian stromal luteoma and helps distinguish it from epithelial, neuroendocrine, melanocytic, and metastatic ovarian tumors.

Based on the combined morphological and immunohistochemical findings, the lesion was diagnosed as an ovarian stromal luteoma, a steroid-producing sex cord-stromal tumor.

Following surgery, the patient’s gonadotropin concentrations rose to postmenopausal levels, estradiol values became consistent with the postmenopausal state, and testosterone concentrations rapidly declined to menopausal levels within a month. As testosterone levels decreased, sex hormone-binding globulin (SHBG) levels increased as expected postoperatively (Table 1). Physical examination at follow-up indicated reversal of virilizing features, including significant reduction in facial hirsutism, stabilization of hair loss, and apparent regrowth in regions of preceding thinning. The patient also reported significant overall improvement (Figure 5).

## 3. Discussion

During the menopausal transition, ovarian function declines, leading to reduced estradiol production and raised gonadotropin levels. Despite the abrupt fall in estrogen, the postmenopausal ovary continues to secrete androgens, which gradually decrease with age. Reduced sex hormone-binding globulin (SHBG) and persistently elevated luteinizing hormone increase the fraction of bioavailable androgens, favoring relative androgen predominance. Mild androgen-dependent changes like facial hair growth or scalp hair thinning could arise from this [14,15,16]. Causes of postmenopausal hyperandrogenism can be broadly categorized as non-tumorous or tumorous. Non-tumorous causes include endocrinopathies such as acromegaly, Cushing disease, hyperprolactinemia, and hyperthyroidism, as well as endogenous disorders like obesity with a PCOS-like phenotype, ovarian hyperthecosis, polycystic ovary syndrome (PCOS), and non-classical congenital adrenal hyperplasia (NCCAH). Exogenous causes include the use of anabolic steroids, certain medications (antiepileptics, glucocorticosteroids), or androgen supplements (testosterone or DHEA). Tumorous causes of postmenopausal hyperandrogenism arise exclusively from the ovaries or the adrenal glands. They should be suspected in women presenting with rapidly progressive symptoms, severe androgen excess, or clinical signs of virilization [17,18]. In postmenopausal women, polycystic ovary syndrome (PCOS) remains the most frequent cause of androgen excess; however, it usually manifests with mild to moderate hyperandrogenic features [5,19]. The appearance of overt virilization, including clitoromegaly, voice deepening, severe hirsutism, and androgenic alopecia, is uncommon and warrants thorough evaluation for an androgen-secreting neoplasm [15,20]. A detailed clinical evaluation was essential in managing our patient. Although the patient had a history of polycystic ovary syndrome (PCOS) during her reproductive years, a direct causal relationship between PCOS and androgen-secreting ovarian tumors has not been established. Nevertheless, a history of PCOS may represent an important diagnostic confounder, as both conditions can present with hyperandrogenic features. In women with previous PCOS, the emergence of atypical features, such as rapidly progressive virilization or marked testosterone elevation, particularly after menopause, should not be attributed solely to the underlying PCOS but should prompt evaluation for an androgen-secreting ovarian or adrenal neoplasm [21,22]. The clinical manifestations of PCOS may change after menopause, but its metabolic and endocrine sequelae may persist in the postmenopausal period. Women with a history of PCOS may continue to exhibit hyperandrogenism, insulin resistance and an adverse metabolic profile after menopause, although the extent of persistent androgen excess varies among individuals. In a Swedish long-term follow-up study, the prevalence of hirsutism was significantly higher in postmenopausal women with PCOS than in control women (33% vs. 4%) at a mean age of 81 years [23].

The patient’s long-term pharmacotherapy was also considered in the differential evaluation of her progressive hyperandrogenism. She had been receiving metformin, a beta-blocker, an angiotensin-converting enzyme (ACE) inhibitor, and a statin for type 2 diabetes mellitus, hypertension, and hyperlipidemia. None of these medications is recognized as a typical cause of marked hyperandrogenism or virilization [15,17]. Metformin is predominantly eliminated unchanged by the kidneys and has a relatively short plasma half-life; importantly, its pharmacodynamic effects on insulin sensitivity and ovarian steroidogenesis are generally associated with a reduction in circulating androgen levels rather than androgen excess [24,25]. In the present case, the patient exhibited significant and rapidly progressive manifestations of hyperandrogenism, including severe hirsutism and virilization, resulting in male-pattern baldness, which is atypical for the usual postmenopausal course of androgen changes. This constellation of findings strongly suggested a hormonally active tumor. The diagnosis of tumor-related androgen excess was further supported by laboratory findings demonstrating inappropriately suppressed gonadotropins and markedly elevated serum testosterone concentrations, reaching levels comparable to those observed in healthy young men. Serum testosterone was 26.8 at baseline and 32.3 during dexamethasone administration. Although this represented a slight numerical increase rather than suppression, the observed difference may reflect analytical assay variability and/or fluctuations in autonomous tumor-associated testosterone secretion rather than a true biological increase induced by dexamethasone. Accordingly, the overall interpretation was that no demonstrable suppression of testosterone secretion occurred during dexamethasone administration [15]. The initial differential diagnosis considered both adrenal and ovarian origins. DHEAS, a unique marker of adrenal zona reticularis activity resulting from sulfotransferase expression, was within the reference range, reducing the likelihood of adrenal involvement [26]. Furthermore, abdominal ultrasonography and magnetic resonance imaging did not reveal any adrenal abnormalities. Androgen-secreting adrenal tumors—adenomas or adrenocortical carcinomas—are usually radiologically detectable and are often associated with clinical or biochemical hypercortisolism. In the absence of these findings, an adrenal source was considered unlikely. Attention subsequently turned to the ovaries. Ovarian tumors may present with subtle alterations in ovarian size or morphology that are frequently overlooked on clinical examination [5]. Transvaginal ultrasonography is a reliable clinical technique for identifying variations in ovarian volume from anticipated age-related standards. Reference values for ovarian volume in various age groups have been established by a number of studies. According to Goswamy et al., women with an average age of 56.3 years had an ovarian volume of 3.57 cm^3^ [27].

In line with these results, Pavlik et al. showed that the average ovarian volume for women between the ages of 50 and 59 was 2.6 ± 0.01 cm^3^, while the average ovarian volume for all postmenopausal women was 2.2 ± 0.01 cm^3^. In postmenopausal women, ovarian volumes larger than 10 cm^3^—approximately two standard deviations above the mean—are typically regarded as abnormal and may suggest a possible cancer, necessitating further diagnostic testing [28]. Initial imaging revealed no adnexal mass, but ovarian volume was not assessed—a critical oversight that likely delayed the detection of the tumor. However, follow-up transvaginal color Doppler ultrasonography performed six months later revealed marked asymmetry in ovarian volume. Ovarian hyperthecosis is typically associated with slowly progressive hyperandrogenism, elevated testosterone (≥5.1 nmol/L measured by LC-MS/MS (Liquid Chromatography–Tandem Mass Spectrometry)), increased gonadotropins, and bilateral ovarian enlargement [14,20]. In our patient, the left ovary measured 11.5 cm^3^, which is unusually large in the postmenopausal setting, despite the absence of a discrete mass or abnormal vascularity. In addition, serum testosterone was markedly elevated at 26.8 nmol/L, as measured by radioimmunoassay, exceeding the threshold for clinically significant androgen excess (>6.9 nmol/L by radioimmunoassay), which corresponds to ≥5.1 nmol/L when assessed by LC–MS/MS. Moreover, gonadotropins were suppressed, and ovarian enlargement was unilateral rather than bilateral. These findings were inconsistent with classic ovarian hyperthecosis and instead suggested an androgen-secreting ovarian tumor, justifying surgical management [14,29].

Combined ovarian and adrenal venous sampling (OVS/AVS) may be considered in selected patients with severe androgen excess when the source of androgen hypersecretion remains uncertain after biochemical evaluation and cross-sectional imaging. This invasive procedure may assist in localizing the source of androgen production by comparing androgen concentrations in adrenal, ovarian, and peripheral venous samples. Its clinical relevance is greatest when identifying a unilateral ovarian source could influence management, particularly in premenopausal women who desire future fertility, as it may allow consideration of unilateral rather than bilateral oophorectomy [30,31].

In our patient, however, the source of androgen excess was already considered highly likely to be ovarian. Normal DHEAS concentrations, together with the absence of adrenal abnormalities on abdominal ultrasonography and magnetic resonance imaging, made an adrenal origin unlikely. Moreover, the markedly elevated serum testosterone concentration and unilateral ovarian enlargement supported an ovarian androgen-secreting tumor. Therefore, additional localization by OVS/AVS was unlikely to provide information that would alter the management course. As our patient was postmenopausal and had completed childbearing, bilateral salpingo-oophorectomy was indicated. Furthermore, OVS/AVS is a technically demanding and invasive procedure requiring advanced expertise, with limited availability outside highly specialized centers; therefore, it was not performed in this clinical setting [14,31].

Histopathological examination ultimately confirmed the presence of a tumor in the left ovary, with immunohistochemical features consistent with a stromal luteoma, which is classified within the group of steroid cell tumors. Ovarian steroid cell tumors (SCTs) represent an uncommon subset of sex cord-stromal neoplasms, accounting for fewer than 0.1% of all ovarian tumors [1,2]. According to the WHO, stromal luteoma and steroid cell tumor not otherwise specified (SCT-NOS) have been subsumed under the broader category of ovarian steroid cell tumors, which are ovarian parenchymal neoplasms composed of steroid-producing cells [1,32]. Steroid cell tumors of the ovary, previously called lipid or lipoid cell tumors, arise from steroid-producing lutein, Leydig, or adrenal-like cells and are traditionally classified as stromal luteoma, Leydig cell tumor, or SCT-NOS. In postmenopausal women, these tumors are often hormonally active, typically causing androgen excess, less frequently leading to estrogenic effects, and rarely to Cushing syndrome [1,2]. Stromal luteomas account for approximately 20–25% of steroid cell tumors. Although they belong to this group, they are generally benign and consist of luteinized steroid cells within the ovarian stroma, reflecting their characteristic histological features. Grossly, these tumors are typically small (<3 cm), with a mean diameter of approximately 1.3 cm, unilateral, well-circumscribed and solid, and characteristically display a gray-white to yellow cut surface on macroscopic examination—features consistent with our observations [3]. In published case series of stromal luteomas, these tumors most often occur in postmenopausal women (80%) and typically present with symptoms attributable to hormone secretion. Although stromal luteomas are usually associated with hyperestrogenism and abnormal uterine bleeding caused by estrogen excess (60% of cases), a minority of patients (approximately 12%) exhibits signs of androgen excess such as hirsutism or virilization. In our case, the patient was referred due to clear hyperandrogenism, which is uncommon but recognized within the spectrum of endocrine activity of these tumors [3,5]. To provide a focused literature review, we performed a targeted PubMed search using combinations of the terms ‘stromal luteoma’, ‘ovarian stromal luteoma’, ‘steroid cell tumor’, and hyperandrogenism-related keywords. Cases reporting estrogen-secreting stromal luteomas, histologically unconfirmed cases, and reports with insufficient information for comparison were excluded. Histologically confirmed cases of androgen-secreting stromal luteoma were included [33,34,35], and the identified reports are summarized in Table 2.

Since the patient had already undergone a total hysterectomy, we could not evaluate clinical features typically associated with estrogen-producing ovarian tumors, such as endometrial hyperplasia, abnormal uterine bleeding, or occasional endometrial adenocarcinoma. Therefore, interpretation of the patient’s estrogen status relied primarily on biochemical findings rather than gynecological manifestations. In postmenopausal women, mild elevations in serum estradiol may also reflect peripheral aromatization of androgens in adipose tissue rather than autonomous ovarian estrogen secretion [36,37]. In the few published reports providing quantitative hormonal measurements in estrogen-secreting steroid cell tumors, serum estradiol concentrations have generally ranged from approximately 56 to 75 pg/mL (≈200–275 pmol/L), values that are clearly above the expected postmenopausal reference range [38,39,40].

Although the patient’s serum estradiol level was slightly elevated for a postmenopausal woman, this finding could be explained by peripheral conversion of androgens to estrogens in adipose tissue rather than by autonomous tumor estrogen secretion. In postmenopausal women, ovarian estrogen production is markedly reduced, and circulating estrogens are predominantly derived from extragonadal sources, particularly adipose tissue, where aromatase converts androgen precursors into estrogens. Importantly, obesity enhances this process, as increased adipose tissue mass is associated with greater peripheral aromatase activity and contributes to higher circulating estrogen concentrations [36,37]. This interpretation is supported by previous studies; a pooled analysis of eight studies demonstrated that postmenopausal women with obesity (BMI ≥ 30 kg/m^2^) had 83% higher circulating total estradiol levels and 60% higher total estrone levels compared with lean postmenopausal women (BMI < 22.5 kg/m^2^), which is consistent with enhanced peripheral aromatization in adipose tissue [41].

In this context, the pre-operative estradiol level observed in our patient (78.8 pmol/L), which decreased following bilateral oophorectomy (21.1 pmol/L), represents a mild and transient elevation. This degree of increase does not support autonomous estrogen secretion by the tumor, which in published cases typically results in substantially higher postmenopausal estradiol concentrations [38,39,40]. Instead, this marginal elevation is most plausibly explained by peripheral aromatization of tumor-derived androgens within adipose tissue, particularly in the setting of obesity [36,37]. Notably, serum androstenedione levels remained within the reference range, arguing against a precursor-driven mechanism of androgen excess and supporting the interpretation of a testosterone-producing tumor.

According to the current WHO Classification of Female Genital Tumours (WHO 2020), stromal luteoma is classified within sex cord-stromal tumors and is diagnosed primarily based on characteristic morphologic features supported by a limited but standardized immunohistochemical panel, typically including inhibin, calretinin, Melan-A, and related markers. Immunohistochemical assessment of steroidogenic enzymes is not currently part of the routine diagnostic evaluation of stromal luteoma and remains largely confined to research settings [7].

In contrast, detailed steroidogenic enzyme profiling has been reported primarily in SCT-NOS, where coordinated expression of CYP11A1, CYP17A1, HSD3B2, AKR1C3, and related enzymes has been associated with androgen excess and virilization [42].

A limitation of our study is that immunohistochemical localization of key steroidogenic enzymes involved in steroidogenesis was not performed despite the availability of formalin-fixed paraffin-embedded tissue blocks. These analyses could not be performed because validated antibodies for steroidogenic enzymes and the additional technical resources required for these exploratory investigations were not available at our institution. Such analyses would have provided valuable mechanistic insight into intratumoral steroidogenesis and could have further clarified the molecular mechanisms underlying testosterone production in this rare tumor.

In the evolving landscape of ovarian tumor classification, the diagnostic designation of our case warrants specific nosological clarification. According to the current 2020 World Health Organization (WHO) Classification of Female Genital Tumours (5th edition), traditional subcategories of steroid cell tumors—specifically stromal luteoma and SCT-NOS—have been broadly subsumed under the single overarching category of ovarian steroid cell tumor (SCT). Under this modern, unified framework, a tumor lacking Reinke crystals that cannot be definitively classified as a Leydig cell tumor is systematically categorized as an SCT-NOS. However, despite this consolidated nomenclature in the newest classification, the clinical, morphological, and topographical features of our patient’s tumor strictly align with the classic criteria for a stromal luteoma as defined in previous classifications. We systematically demonstrated several key characteristics that definitively substantiate this specific diagnosis: (1) Size and circumscription—the tumor was a small (1.5 cm), solitary and well-circumscribed, and was entirely confined within the ovarian parenchyma. (2) Stromal topography—histopathological mapping confirmed that the nodule was distinctly located within the ovarian stroma and was completely independent of the ovarian hilum, effectively ruling out a hilus/Leydig cell tumor origin. (3) Absence of Reinke crystals—no Reinke crystals were identified upon examination of the H&E-stained sections. Together with the non-hilar location, this finding further distinguishes it from a typical hilar Leydig cell tumor. (4) Absence of background hyperthecosis—extensive examination of the available tissue confirmed the absence of diffuse or multifocal stromal hyperthecosis in the surrounding non-neoplastic ovarian parenchyma. Although stromal hyperthecosis is a characteristic and frequent finding in stromal luteoma, it is not an obligatory. In the original clinicopathological series by Hayes and Scully [43] stromal hyperthecosis was identified in 92% of cases, indicating that a minority of stromal luteomas may occur without demonstrable hyperthecosis [4]. Therefore, the absence of hyperthecosis in our case does not preclude the original diagnosis of stromal luteoma. This finding also helps distinguish the lesion from a nodular focus of stromal hyperthecosis. By confirming this precise constellation of microscopic and gross findings, our case successfully demonstrated the specific diagnostic features of a stromal luteoma. Despite its modern grouping under the broader SCT-NOS umbrella, it is clinically and scientifically vital to highlight this specific designation. This terminology precisely reflects the benign nature and distinct parenchymal pathogenesis of this rare, androgen-secreting neoplasm [7].

In postmenopausal women with virilization and an ovarian source of androgen excess, surgery is the treatment of choice. As fertility preservation is not required, laparoscopic bilateral oophorectomy with or without hysterectomy is recommended [14]. Given our patient’s prior hysterectomy, bilateral salpingo-oophorectomy was performed to remove the androgen source and confirm the diagnosis. Stromal luteomas are typically benign and adequately treated with oophorectomy alone [5]. Postoperative hormonal follow-up, as in our patient, is essential, as testosterone levels normalize rapidly, whereas clinical signs of virilization improve gradually [14,44].

## 4. Conclusions

This case highlights the need to consider rare androgen-secreting ovarian tumors in postmenopausal women presenting with rapid virilization and markedly elevated testosterone, even when initial imaging shows no masses. Assessing ovarian volume alongside routine imaging is critical. Careful hormonal evaluation and repeat targeted imaging were essential to identify the small ovarian steroid cell tumor. Surgical removal provided a definitive diagnosis and resolved both biochemical abnormalities and clinical manifestations. Early recognition and thorough evaluation are key to preventing delayed diagnosis and prolonged androgen excess.

## Figures and Tables

**Figure 1:**
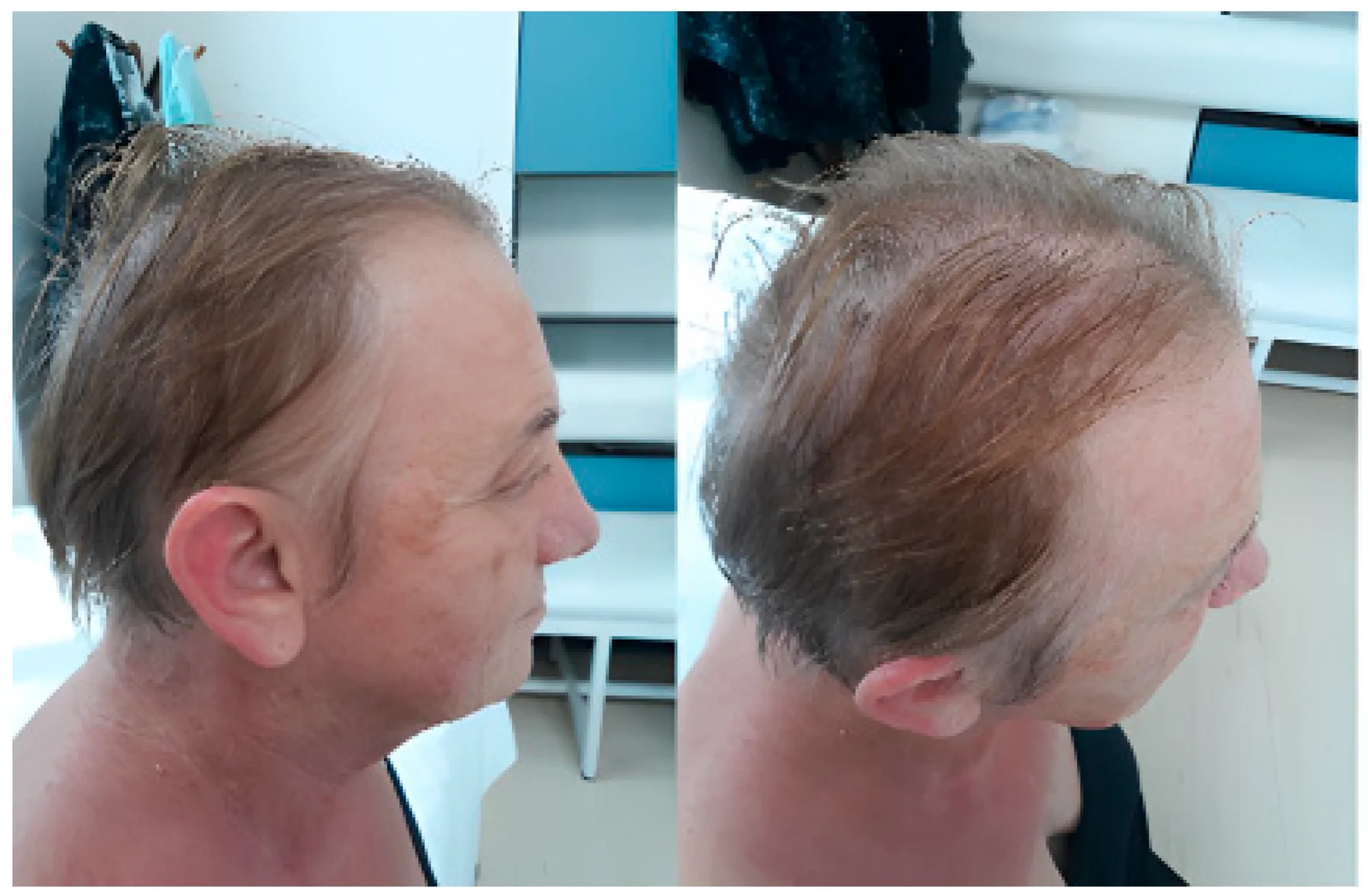
Clinical image: Androgen alopecia.

**Figure 2:**
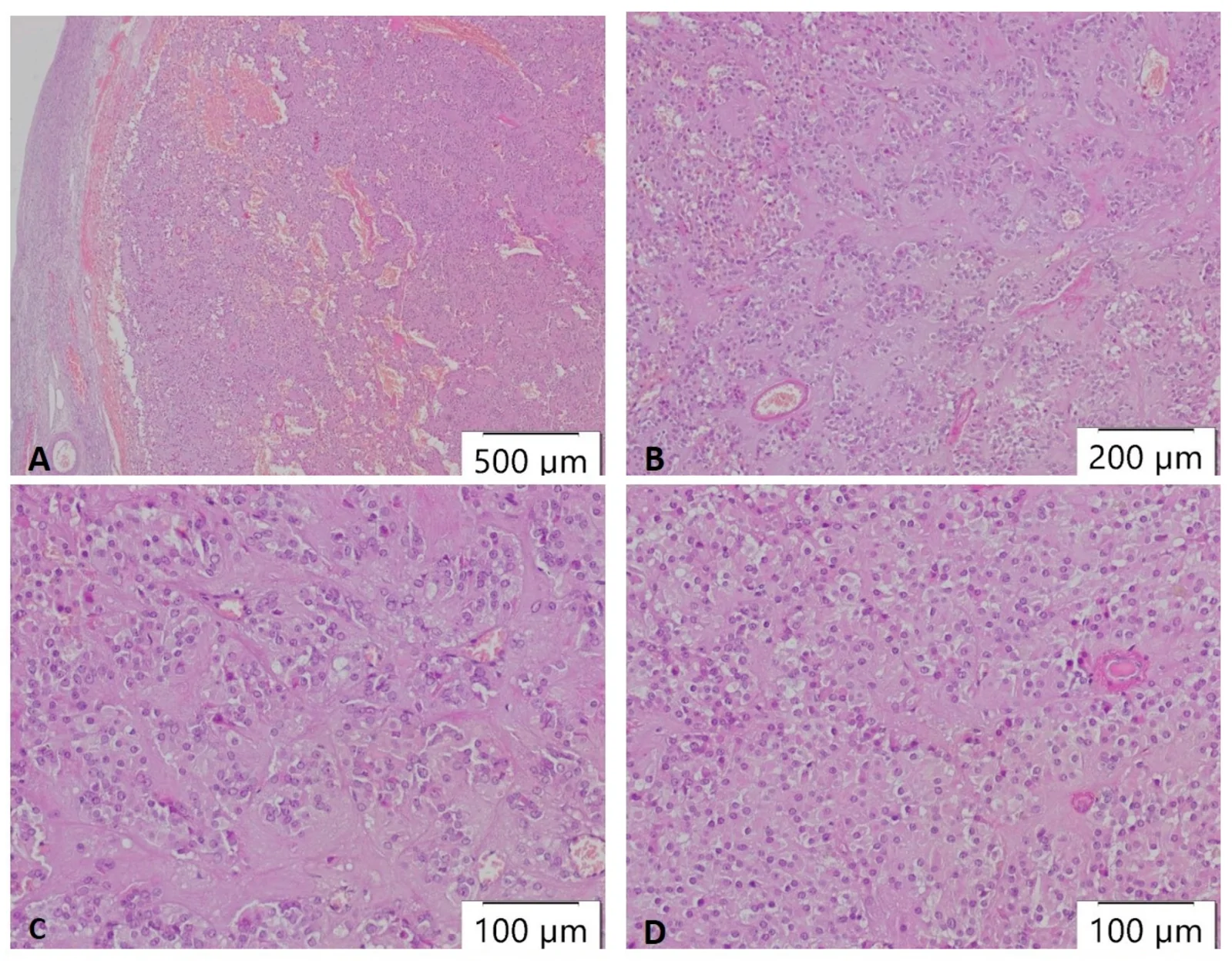
Ovarian tumor nodule. (**A**) Hematoxylin–eosin stain, magnification ×40; (**B**) hematoxylin–eosin stain, magnification ×100; (**C**,**D**) hematoxylin–eosin stain, magnification ×200, showing tumor composed of luteinized-appearing cells with areas of hemorrhage.

**Figure 3:**
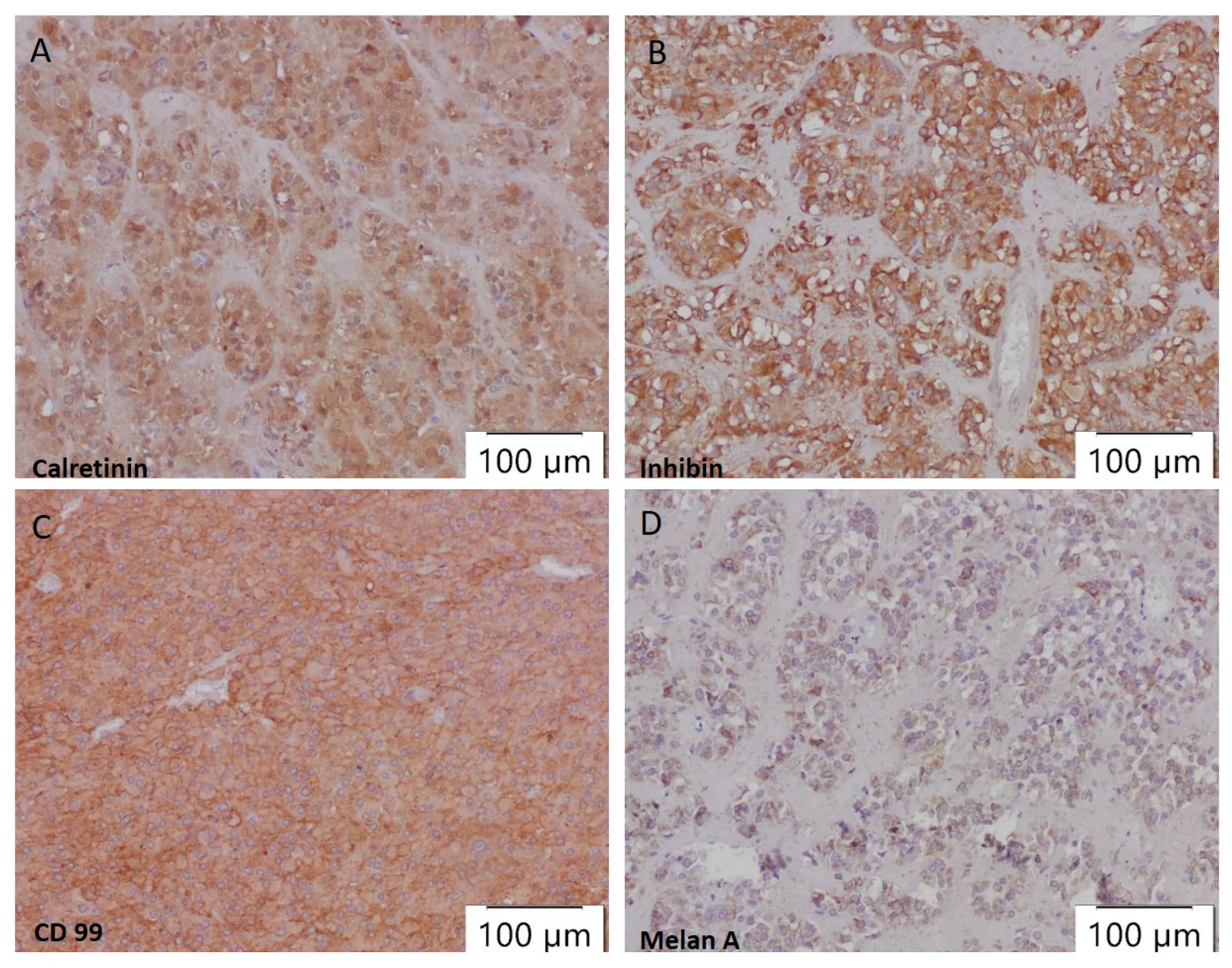
Immunohistochemical positivity in the ovarian tumor: (**A**) Calretinin, ×200; (**B**) Inhibin, ×200; (**C**) CD99, ×200; (**D**) Melan-A, ×200.

**Figure 4:**
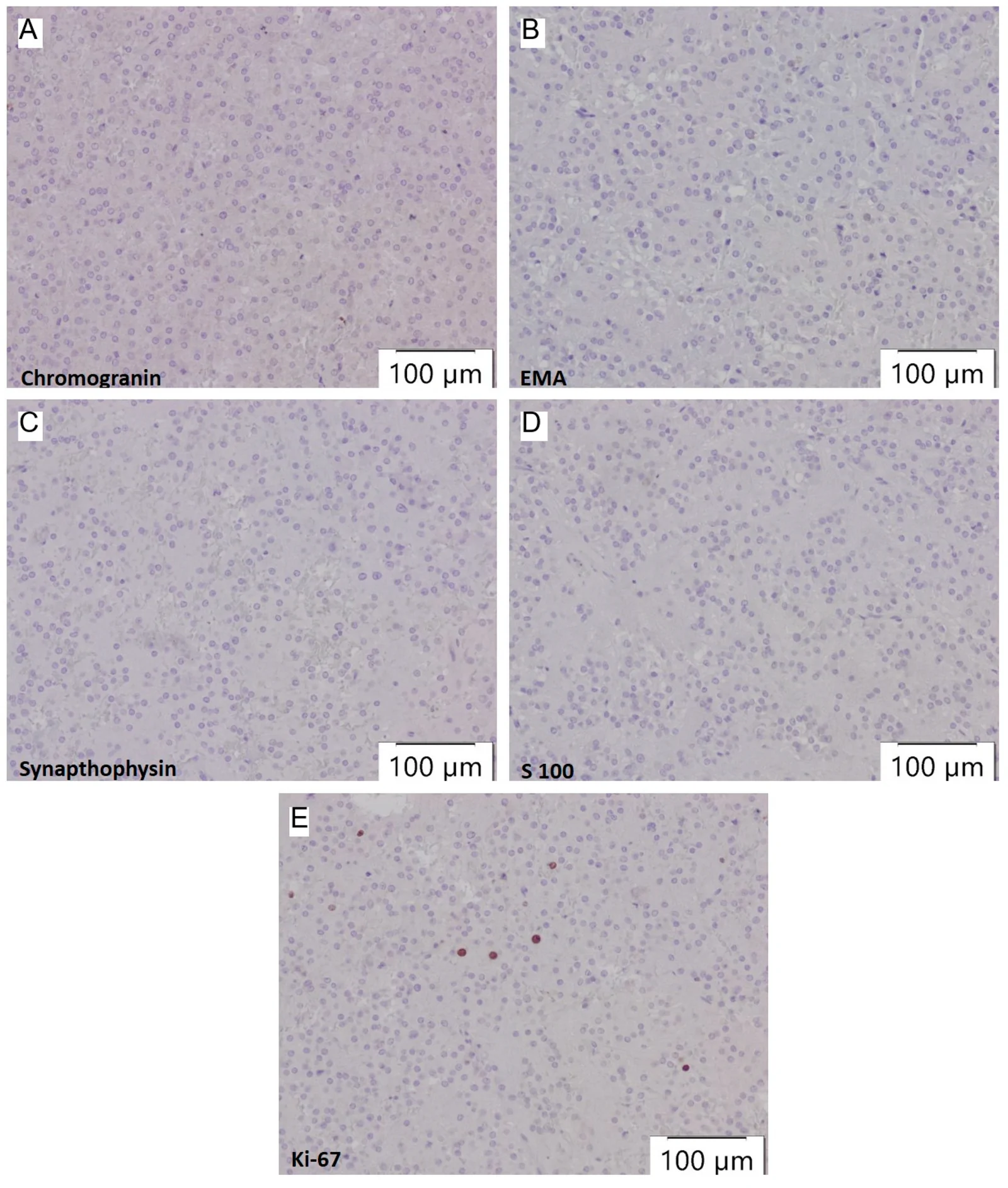
Immunohistochemical negativity in the ovarian tumor: (**A**) Chromogranin, ×200; (**B**) EMA, ×200; (**C**) Synaptophysin, ×200; (**D**) S-100 protein, ×200; (**E**) Ki-67, ×200.

**Figure 5:**
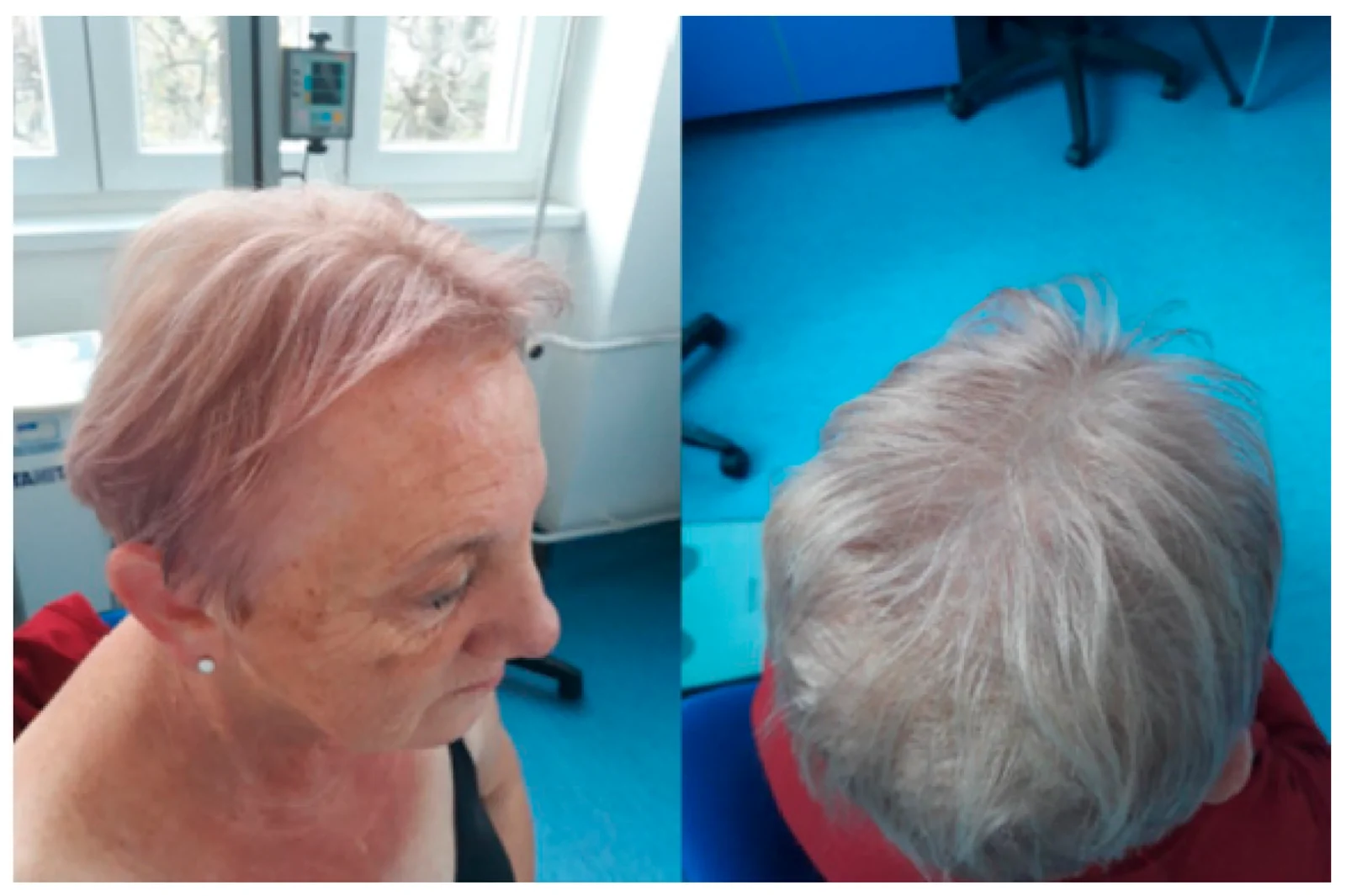
Clinical image 8 months after surgical removal of an androgen-producing ovarian stromal luteoma: regression of androgenic alopecia.

**Table 1:** Hormonal analysis before and one month after surgery.

	Reference Value	Basal Values Before Surgery	LDDST Before Surgery	Basal Values After Surgery
FSH, IU/L	>40 (menopause)	3.5		30.4
LH, IU/L	>40 (menopause)	3.8		20.4
Estradiol, pmol/L	<50 (menopause)	78.8		21.1
LTH, mIU/L	<550	265		
17-OHP, nmol/L	0.303–2.42	1.6	2.3	1.7
Testosterone, nmol/L	0.3–3	26.8	32.3	0.5
SHBG, nmol/L	18–87	26.9		66.4
FAI	7–10	99.6		0.7
DHEAS, pmol/L	1.9–10.5	1.5	0.98	1.7
Androstenedione, ng/mL	0.02–2.26	1.5	0.88	0.8
Inhibin B, pg/mL	<10	5.7		
AMH, ng/mL	<0.01 (menopause)	<0.01		
ACTH, ng/L	10–90	17.6		12.3
Cortisol, nmol/L	131–642	260.6	20.5	510.2

FSH = Follicle-stimulating hormone, LH = Luteinizing hormone, LTH = prolactin, 17-OHP = 17-Hydroxyprogesterone, SHBG = Sex hormone-binding globulin, FAI = Free androgen index, DHEAS = Dehydroepiandrosterone sulfate, AMH = Anti-Mullerian hormone, ACTH = Adrenocorticotropic hormone.

**Table 2:** Reported cases of androgen-secreting ovarian stromal luteomas.

Author, Year	Age/Parity Status	Clinical Presentation	Testosterone Level	Tumor Size	Imaging Findings	Outcome
Ajith, 2016 [33]	70 y, nulliparous	Gradual hyperandrogenism (FG score 20, 10 years), hirsutism, alopecia	8 nmol/L (NR not reported)	NA	Normal adrenal imaging	Symptom and hormonal improvement after laparoscopic BSO; no recurrence (2 years)
Ajith, 2016 [33]	63 y, multiparous	Gradual hyperandrogenism (FG score 18, 5 years)	7.5 nmol/L (NR not reported)	~0.5 cm grayish-brown lesion	NA	Post-op improvement; no recurrence (2 years)
Bogdanou, 2016 [5]	55 y, primiparous	Severe virilization (FG 23–26): hirsutism, alopecia, deep voice, clitoromegaly	Total testosterone 775 ng/dL (NR 2.9–40.8 ng/dL); free testosterone 116.42 ng/dL (NR 53.5)	20 × 20 mm brown ovarian nodule	CT/MRI/EUS: no adrenal or ovarian tumor identified	Testosterone normalized 1 week post-op; clinical improvement
Chico, 1995 [34]	64 y	Progressive virilization (FG 18), hirsutism, alopecia, clitoromegaly	17 nmol/L (NR 0.3–3 nmol/L)	1.1 × 1.0 cm yellow-brown cortical nodule	CT: mild adrenal enlargement, otherwise normal	Post-op normalization of androgens; symptom regression
Honore, 1992 [35]	76 y	Long-standing hirsutism (15 years), alopecia, clitoromegaly	5.1 nmol/L (NR 0.5–1.8 nmol/L)	10 × 8 × 8 mm dark brown corticomedullary tumor	NA	Post-op normalization of testosterone; regression of virilization; no recurrence (3 years)

FG score: Ferriman–Gallwey score; NA: Not available; NR: Normal range; CT: Computed tomography; MRI: Magnetic resonance imaging; EUS: Endoscopic ultrasound; Post-op: Postoperative; BSO: Bilateral salpingo-oophorectomy.

## Data Availability

The original contributions presented in this study are included in the article. Further inquiries can be directed to the corresponding author. This does not imply that identifiable patient records will be publicly released.

## Abbreviations

The following abbreviations are used in this manuscript:

WHO	World Health Organization
SCTs	Steroid cell tumors
PCOS	Polycystic ovarian syndrome
ACE	Angiotensin-converting enzyme
BMI	Body mass index
DHEAS	Dehydroepiandrosterone sulfate
FSH	Follicle-stimulating hormone
LH	Luteinizing hormone
LTH	Prolactin
17-OHP	17-Hydroxyprogesterone
FAI	Free androgen index
AMH	Anti-Mullerian hormone
ACTH	Adrenocorticotropic hormone
LDDST	Low-dose dexamethasone suppression test
TSH	Thyroid-stimulating hormone
fT4	Free thyroxine
CEA	Carcinoembryonic antigen
CA-125	Cancer antigen-125
HE4	Human epididymis protein 4
CA-19-9	Carbohydrate antigen 19-9
AFP	Alpha-fetoprotein
NSE	Neuron-specific enolase
β-hCG	Beta-human chorionic gonadotrophin
MRI	Magnetic resonance imaging
MART-1	Melanoma antigen recognized by T-cells
H&E	Hematoxylin and eosin
CD99	Cluster of differentiation 99
EMA	Epithelial membrane antigen
SHBG	Sex hormone-binding globulin
NCCAH	Non-classical congenital adrenal hyperplasia
DHEA	Dehydroepiandrosterone
LC–MS/MS	Liquid chromatography–tandem mass spectrometry
SCT-NOS	Steroid cell tumor not otherwise specified
OVS/AVS	Ovarian venous sampling/Adrenal venous sampling
CT	Computed tomography
EUS	Endoscopic ultrasound
BSO	Bilateral salpingo-oophorectomy
Post-op	Postoperative
CYP11A1	Cytochrome P450 family 11 subfamily A member 1
CYP17A1	Cytochrome P450 family 17 subfamily A member 1
HSD3B2	Hydroxy-delta-5-steroid dehydrogenase, 3 beta- and steroid delta-isomerase 2
AKR1C3	Aldo-keto reductase family 1 member C3
